# Notch Signaling in Myeloid Cells as a Regulator of Tumor Immune Responses

**DOI:** 10.3389/fimmu.2018.01288

**Published:** 2018-06-04

**Authors:** Fokhrul Hossain, Samarpan Majumder, Deniz A. Ucar, Paulo C. Rodriguez, Todd E. Golde, Lisa M. Minter, Barbara A. Osborne, Lucio Miele

**Affiliations:** ^1^Department of Genetics, Louisiana State University Health Sciences Center, New Orleans, LA, United States; ^2^Stanley S. Scott Cancer Center, Louisiana State University Health Sciences Center, New Orleans, LA, United States; ^3^H. Lee Moffitt Comprehensive Cancer Center, Tampa, FL, United States; ^4^Department of Neurosciences, McKnight Brain Institute, University of Florida at Gainesville, Gainesville, FL, United States; ^5^Department of Veterinary and Animal Sciences, University of Massachusetts, Amherst, MA, United States

**Keywords:** Notch, cancer, immunity, cellular, inflammation, myeloid cells

## Abstract

Cancer immunotherapy, which stimulates or augments host immune responses to treat malignancies, is the latest development in the rapidly advancing field of cancer immunology. The basic principles of immunotherapies are either to enhance the functions of specific components of the immune system or to neutralize immune-suppressive signals produced by cancer cells or tumor microenvironment cells. When successful, these approaches translate into long-term survival for patients. However, durable responses are only seen in a subset of patients and so far, only in some cancer types. As for other cancer treatments, resistance to immunotherapy can also develop. Numerous research groups are trying to understand why immunotherapy is effective in some patients but not others and to develop strategies to enhance the effectiveness of immunotherapy. The Notch signaling pathway is involved in many aspects of tumor biology, from angiogenesis to cancer stem cell maintenance to tumor immunity. The role of Notch in the development and modulation of the immune response is complex, involving an intricate crosstalk between antigen-presenting cells, T-cell subpopulations, cancer cells, and other components of the tumor microenvironment. Elegant studies have shown that Notch is a central mediator of tumor-induced T-cell anergy and that activation of Notch1 in CD8 T-cells enhances cancer immunotherapy. Tumor-infiltrating myeloid cells, including myeloid-derived suppressor cells, altered dendritic cells, and tumor-associated macrophages along with regulatory T cells, are major obstacles to the development of successful cancer immunotherapies. In this article, we focus on the roles of Notch signaling in modulating tumor-infiltrating myeloid cells and discuss implications for therapeutic strategies that modulate Notch signaling to enhance cancer immunotherapy.

## Introduction

Notch signaling, an evolutionarily conserved cell-fate-determination pathway, mediates close contact interactions between neighboring cells. Notch is involved in many aspects of tumor biology, from angiogenesis to cancer stem cells maintenance to tumor immunity (1–3). Mammals have four structurally related Notch receptors (Notch1–4) that bind transmembrane ligands of the Jagged (Jagged-1, Jagged-2) or the Delta-like (DLL1, DLL3, and DLL4) families (2, 4, 5). Binding of Notch receptors to ligands, or in some cases, ligand-independent receptor activation (6) triggers separation of the extracellular receptor subunit from the transmembrane subunit. The latter undergoes a multistep proteolytic process, which results in the release of a Notch intracellular domain (NICD) (7). NICD translocate into the nucleus and complexes with the CSL (CBF-1/Suppressor of Hairless/LAG-1, also known as RBP-J), and mastermind-like (MAML1-3) coactivator and other proteins to form the Notch transcriptional complex, which regulates the transcription of multiple genes (2, 4, 5, 7). In addition to canonical Notch signaling, several non-canonical (CSL-independent) Notch signals have been described in oncogenesis and inflammation (8–10). Context-dependent Notch signaling regulates many cell fate choices and Notch dysregulation contributes to the development of various malignancies (5). Notch signaling can produce different biological outcomes depending on the timing and the strength of the signals as well as the expression of different ligand/receptor pairs, post-translational modifications, or receptors and specific regulation at both the transcriptional and post-transcriptional level (11, 12). Hyperactivation of Notch has been considered as oncogenic in several cancers including breast cancer and lymphoid malignancies (T-cell acute lymphoblastic leukemia, T-ALL, B-chronic lymphocytic leukemia, and splenic marginal zone lymphoma). On the other hand, loss of function of individual Notch paralogs has revealed tumor-suppressive activities in other malignancies, as reviewed in Ref. (13, 14).

Myeloid cells are essential for the homeostasis of the innate and adaptive immune responses. Myeloid cells [granulocytes, macrophages, and dendritic cells (DCs)] develop from hematopoietic stem cells (HSCs) through sequential differentiation steps under normal physiological conditions. However, multiple soluble factors released by the tumor microenvironment (both tumor cells and tumor-associated stromal cells) perturb the normal myeloid development resulting in the accumulation of myeloid-derived suppressor cells (MDSCs), a heterogeneous group of immature myeloid cells with immune-suppressive properties. In addition, tumor-derived soluble factors induce defects in the differentiation of DCs and accumulation and polarization of tumor-associated macrophages (TAMs), as described in Ref. (15). Although the importance of Notch signaling in myeloid cells differentiation is well understood, the exact nature of Notch effects remains controversial. There is literature supporting a critical role of Notch in the maintenance of progenitor cells to delay the terminal differentiation of myeloid cells, while other data suggest that Notch signaling is required for differentiation of mature myeloid cells, as reviewed in Ref. (16). Overall, it is probably fair to say that the role of Notch signaling in myeloid cell differentiation is context dependent; it depends on the timing of Notch activation and the differentiation stages of myeloid cells.

T-cell-based cancer immunotherapy has shown effectiveness in some highly lethal malignancies and offers a great deal of promise for the treatment of others. Although the Food and Drug Administration (FDA) approved few T cell-based immunotherapy agents and several others are in phase I–II clinical trials, clinical outcomes have not been as universally positive as initially thought. The presence of a tolerogenic microenvironment that blocks the antitumor effector functions of T cells is a major factor limiting the clinical efficacy of T-cell-based immunotherapy (17). Tumor-infiltrating myeloid cells are central components of the tolerogenic tumor microenvironment, along with regulatory T cells (T_regs_). Recently, Campese et al. described a role of Notch in immunoregulatory cells including T_reg_ in the context of tumor microenvironment (18). In this review, we will discuss the role of Notch signaling in myeloid cells (MDSC, DC, and macrophages) as a modulator of tumor immune response.

## Notch and MDSC

Myeloid-derived suppressor cells are major immune response regulators in cancer and other pathological conditions. MDSCs are a heterogeneous population of cells consisting of myeloid progenitor cells and immature myeloid cells that have immune-suppressive functions, as reviewed in Ref. (19). MDSCs adversely modulate the immune response to cancer and also facilitate tumor metastasis and angiogenesis (15, 19, 20). The immune-suppressive function of MDSC is mediated through the expression of arginase1 (ARG1), inducible NOS, formation of peroxynitrite, expression of TGF-β, IL10, and COX2, sequestration of cysteine, and induction of immunosuppressive T_regs_, among others, as reviewed in Ref. (15, 21). In mice, MDSCs are defined by the co-expression of CD11b and Gr-1 markers and consist of two major subsets, the granulocytic polymorphonuclear (PMN)-MDSC (CD11b^+^Ly6G^+^Ly6C^lo^) and the M-MDSC (CD11b^+^Ly6G^−^Ly6C^hi^) (22). However, in humans, the situation appears to be more complex, and several different markers of MDSCs have been described (22).

Although the role of Notch signaling in myelopoiesis remains somewhat controversial, a number of studies have demonstrated that Notch signaling is important for the accumulation of MDSC (18, 23, 24). Transgenic mice that overexpress ADAM10 (responsible for the first proteolytic cleavage of Notch transmembrane subunits) resulted in abrogated B cell development, delayed T cell development in the thymus but systemic expansion of CD11b^+^Gr1^+^ MDSC (25). Gibb et al. (25) suggested that differential cleavage of Notch1 into S2 and S3 products modulated by ADAM10 is important to hematopoietic cell-fate determination. Notch was shown to induce myeloid differentiation of multipotent hemopoietic progenitor cells by upregulating the expression of the transcription factor PU.1, suggesting that Notch signaling functions as an extrinsic regulator of myeloid commitment (26).

Gabrilovich et al. reported that inhibition of Notch signaling in hematopoietic progenitor cells (HPCs), MDSCs, and DCs correlates with abnormal myeloid cell differentiation in cancer (23). The inhibition of Notch signaling in these cells is mediated by NICD phosphorylation by casein kinase 2, which disrupts the interaction between NICD and CSL. Another group (27) also reported that blockade of Notch signaling induced the generation of PMN-MDSC with lower immunosuppressive function, but inhibited the production of mononuclear-MDSC. They also showed that Notch-CSL signals modulate the differentiation process and immunosuppressive functions of MDSC. One possible mechanism whereby Notch signaling could regulate MDSC differentiation is through miR-223. Notch suppresses miR-223 expression in rheumatoid arthritis macrophages (28). In turn, miR-223 inhibits the differentiation of tumor-induced MDSC (29), regulating their number and immune-suppressive functions (30).

Myeloid-derived suppressor cells within the tumor microenvironment block the effects of adoptive T cell-based immunotherapy (ACT) by inhibiting several T cell functions, including T cell proliferation and the expression of various cytotoxic mediators. The success of ACT depends on differentiation of CD8^+^ T cells into cytolytic and cytokine-producing effector cells (31). However, limited exposure to MDSCs can paradoxically enhance the effectiveness of ACT. Acquisition of full effector function *in vitro* impairs the antitumor efficacy of CD8^+^ T cell-based ACT (32). In fact, transfer of activated stem cell memory T cells resulted in higher antitumor responses in mice than effector memory T cells (33). These results suggest that inhibition of CD8^+^ cell differentiation can enhance the antitumor activity of CD8^+^ T cells following ACT. Rodriguez et al. (34) reported that transient conditioning of CD8^+^ T cells with MDSC blocks their differentiations into effector T cells and significantly improves their antitumor activity following ACT. Their results indicated that conditioning of T cells with MDSC induces stress survival pathways through blunted mTOR signaling, which in turn modulated T cell differentiation and ACT efficacy. Thus, short-term conditioning T cells with MDSC could prove beneficial in ACT strategies for cancer immunotherapy.

An elegant study by Peng et al. (35) suggested that the presence of MDSC in tumors is correlated with the presence of cancer stem-like cells (CSCs) and both independently predict poor patient survival. These authors suggested that MDSC-derived IL-6 and nitric oxide (NO) may collaborate to activate STAT3 and Notch signaling and induce breast CSCs. Notch signaling has also been proposed to induce cancer metastasis by promoting the migration of MDSCs. Nakayama et al. reported that F-box protein FBXW7 has tumor-suppressive capacity and inhibits cancer metastasis (36). FBXW7 is an E3 ubiquitin protein ligase involved in the degradation of several oncoproteins including NICD. Deletion of Fbxw7 in murine bone marrow-derived stromal cells resulted in the accumulation of Notch1 and increased expression of CCL2. CCL2 in turn facilitated the recruitment of M-MDSC and macrophages, promoting metastatic tumor growth.

The role of Notch in T cell-mediated cancer immunity has been studied extensively (8, 37). Rodriguez et al. (38) reported that the tumor microenvironment suppresses Notch1 and Notch2 expression in CD8 T cells. Conditional expression of transgenic Notch1 intracellular domain (N1ICD) in activated antigen-specific CD8^+^ T cells induced cytotoxic responses and caused CD8^+^ T cells to become resistant to MDSC-mediated tolerogenic effects in tumor-bearing mice (38). MDSC blocked the expression of Notch in T cells *via* NO-dependent mechanisms. The authors suggested that transgenic expression of Notch1 or Notch2 NICD in CD8^+^ T cells or chimeric antigen receptor T (CAR-T) cells may overcome MDSC-mediated tolerogenic effects and prove therapeutically beneficial. However, the molecular mechanisms whereby MDSC-derived NO inhibits Notch signaling remain unclear.

Recently, the Rodriguez lab in collaboration with the Miele and Osborne labs showed that tumor MDSC, unlike circulating MDSC, upregulate expression of Notch ligand Jagged1, and to a lesser extent, Jagged2. This phenomenon is mediated by NF-κB (39). Treatment with an anti-Jagged1/2-blocking antibody had remarkable therapeutic activity in several mouse models (3LL lung carcinoma and EG-7, an ovalbumin-expressing form of EL-4 lymphoma), which depended upon enhanced CD8 responses (39). In EG-7 tumors, anti-Jagged antibodies enhanced the effect of anti-ovalbumin adoptive T-cell therapy (ACT). Interestingly, anti-Jagged therapy induces the appearance of potentially immune-stimulatory MDSC-like cells (MDSC-LC), which had lower expression of MDSC-suppressive mediators, iNOS and ARG1. It is unclear whether these MDSC-LC derive from the reprogramming of MDSC or from *de novo* differentiation from bone marrow myeloid precursors upon Jagged inhibition. It is also unclear how Jagged blockade produces this effect. It may allow DLL ligands to activate Notch with a different kinetics, or possibly relieve *cis*-inhibition of MDSC Notch receptors by Jagged ligands expressed on the same cells. Further mechanistic investigations are necessary to answer these questions. However, these findings provide a preclinical proof of concept for the use of anti-Jagged1/2 antibodies to reprogram MDSC-mediated T-cell suppression to enhance the efficacy of cancer immunotherapy.

In summary, the Notch pathway can be considered a multifaceted modulator of MDSC biology. Notch signals modulate MDSC activity in different ways, depending on the receptors and ligands involved, microenvironmental clues (e.g., NF-κB activation by inflammatory cytokines), the stages of myeloid cells differentiation, as well as the subpopulation of cells. Targeting Jagged-family Notch ligands to inhibit MDSC is a promising strategy to overcome tumor tolerance.

## Notch and DCs

Dendritic cells are professional antigen-presenting cells (APC) that recognize, acquire, process, and present antigens to resting T cells to activate antigen-specific immune responses. The engagement of DC in the induction of immune responses against a myriad of pathogens, tumor cells, and self-antigens is a cornerstone of adaptive immunity (40). DCs include distinct functional subsets including interferon-producing plasmacytoid DCs (pDCs) and classical DCs (myeloid) (41–43). Classical DCs are the dominant subset and differentiate along the myeloid lineage pathway. The mechanisms of differentiation of these two subsets are vastly different, although they converge on some pathways (41–43). Decreased DC function has been suggested as a major cause of the observed defect in cell-mediated immunity in patients with advanced breast cancer (44). DC differentiation from HPCs is controlled both by a network of soluble growth factors and cytokines produced by bone marrow stroma (BMS) and direct cell–cell contact with BMS *via* a complex network of soluble factors and cell-bound molecules. Several studies have implicated Notch signaling in DC differentiation and function (45–47).

There is both consensus and controversy surrounding the extent of Notch involvement in DC differentiation. Several groups have described a direct role of Notch in promoting DC differentiation. Expression of DLL1 in conjunction with GM-CSF induced differentiation of bone marrow cells to DCs at the expense of other lineages (48). In “emergency myelopoiesis,” DLL1 promoted DC differentiation while Jagged1 inhibited it. Both ligands activated Notch, but DLL1 also induced Wnt while Jagged suppressed it by inhibiting the expression of Wnt receptor Frizzled (49). Cheng et al. (50) showed that differentiation of DC was severely compromised in Notch1 antisense mice that have about half the physiological level of Notch1 in HPC. These findings were confirmed in an experimental model of DC differentiation from embryonic stem (ES) cells. Notch1^−/−^ ES are unable to differentiate into DC. In this model, Notch signaling is necessary but not sufficient for DC differentiation (45). On the other hand, Radtke et al. (51) generated *Notch1* conditional knockout mice using the Cre-Lox system and demonstrated that the number of thymic DCs, conventional DCs, and Langerhans cells were normal. Whether other Notch paralogs can compensate for Notch1 deficiency in this model is unclear. Conditional deletion of CSL (RBP-Jκ), which abrogates all canonical Notch signaling in BM cells and DCs resulted in substantial reduction in the presence of conventional DCs in spleens of the knockout mice (52). This decrease affected primarily the CD8^−^ DC subset in the spleen marginal zone (52). Weijzen et al. (46) demonstrated that peptides from the DSL (Delta-Serrate-LAG1) receptor-binding region of Jagged1 promote the maturation of monocytes into myeloid DC. This effect may be mediated by direct activation of Notch receptors or relief of *cis*-inhibition by endogenous Jagged ligands. Lewis et al. (53) demonstrated that Notch2 is required for the functional differentiation of DCs in the spleen and intestine. De Smedt et al. (54) demonstrated an exquisite dose dependence of Notch signaling in the thymic microenvironment, with different levels of Notch signal intensity biasing cell fate decisions toward NK, B, DC, macrophage, or T cell lineages.

Similar contradictory data exist in the literature with respect to the role of Notch signaling in pDCs. It was reported that Notch signaling *via* DLL1 prevents the differentiation of pDC from early thymocyte precursors by decreasing expression of ETS transcription factor Spi-B. Conversely, Jagged1 did not suppress Spi-B expression. Stromal cells expressing DLL1 blocked pDC development (55). However, in a different study, Notch1^−/−^ bone marrow precursors developed normally into thymic pDC, suggesting that thymocytes and pDC originate from different lineages and that Notch only modulates the thymocyte lineage (56).

There is emerging evidence of crosstalk between Notch and Wnt pathways in the regulation of DC differentiation (57). Inhibition of Notch signaling can lead to accelerated differentiation of HSCs *in vitro* and depletion of HSCs *in vivo* (57). Regulation of Notch signaling by the Wnt pathway also plays a vital role in differentiation of precursors along T or NK differentiation pathways (58). Table 1 summarizes some of the key findings reported on the role of Notch signaling in the differentiation and function of tumor-associated myeloid cells.

**Table 1:** Notch effects in the differentiation and function of tumor-associated myeloid cells.

Cell population	Observation	Reference
Dendritic cell	Notch signaling induces differentiation	(15, 16, 40)
Hematopoietic progenitor cell (HPC)	Notch signaling promotes NF-κB-dependent differentiation of HPC	(50)
Macrophages	Notch signaling mediators are upregulated in activated macrophages	(77–80)
Macrophages	DLL4-induced Notch signaling mediates inflammatory responses	(76)
Tumor-associated macrophages (TAMs)	Notch signaling modulates the M1 versus M2 macrophages polarization in antitumor immune response. M2-like TAMs have decreased Notch activity	(81)
Myeloid-derived suppressor cell (MDSC)	Notch signaling is important for the accumulation of MDSC	(18, 24)
MDSC	Notch signaling induces multilineage myeloid differentiation	(26)
MDSC	Blockage of Notch signaling promotes MDSC generation	(23, 27)
MDSC	Anti-jagged therapy to reprogram MDSC by relieving Notch inhibition	(39)

**Figure 1:**
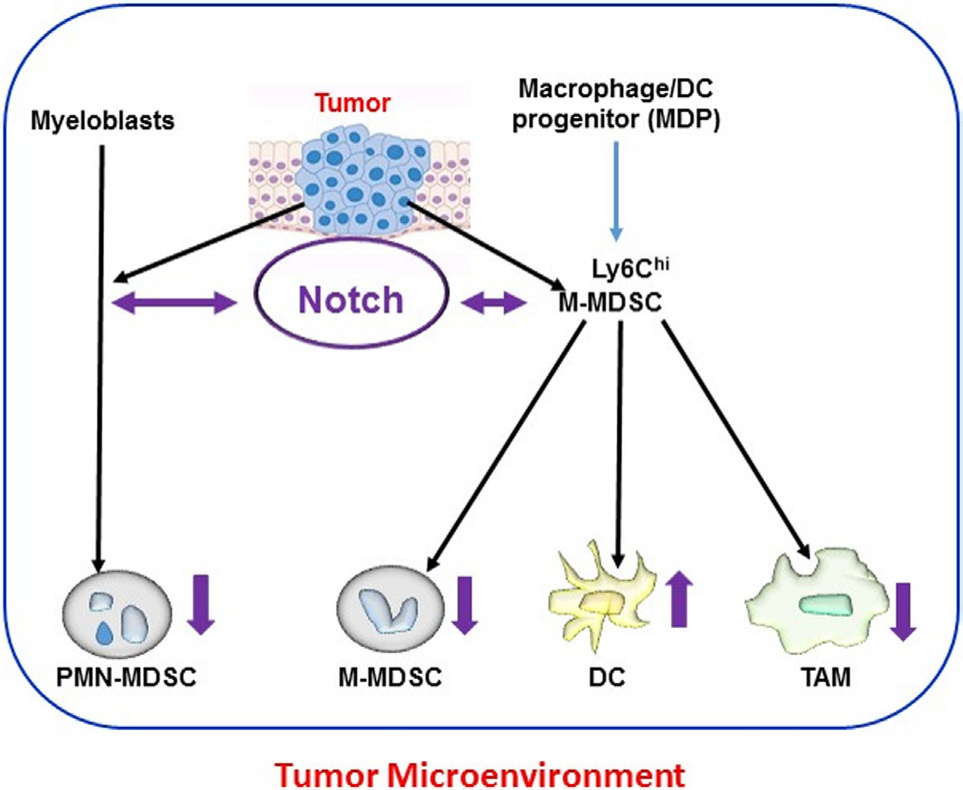
Notch and myeloid cells differentiation within tumor microenvironments. Myeloid cells [polymorphonuclear (PMN) cells, dendritic cells (DCs), and macrophages] derive from hematopoietic stem cells through common myeloid progenitors and the granulocyte-myeloid progenitors lineage. In tumor-bearing hosts, this differentiation process is altered by tumor-derived signals. Expansion of activated PMN-MDSC and M-MDSC occurs from myeloblasts and M-MDSC, respectively, during tumorigenesis. M-MDSC also differentiate into tumor-associated macrophages (TAMs) and DC at the tumor site. Notch signaling mediates bidirectional crosstalk at multiple steps of myeloid cells differentiation in the tumor microenvironment. Differential Notch expression and activity (as indicated by the direction of purple arrows) has been reported in different myeloid populations, with lower Notch expression in myeloid-derived suppressor cell (MDSC) and TAM and higher expression in DC.

These findings highlight two general features of Notch signaling, namely, its context dependence and dose dependence. Notch signals do not appear to operate as an on/off switch. Rather, in many systems, these signals appear to operate based on an intensity gradient that modulates and is modulated by other pathways. Complete blockade of Notch signals is not always necessary to change cellular phenotypes, and small variations in signal intensity or duration may have major phenotypic consequences. Figure 1 schematically depicts the current consensus on the role of Notch signaling in the differentiation and function of tumor microenvironment-associated myeloid cells.

Notch signals are involved not only in the maturation of DC but also in their effector functions. DCs express both Notch receptors and ligands as well as toll-like receptors (TLRs) (59). TLRs potently stimulate the expression of Notch ligands in DC (59). TLRs are being increasingly adopted in DC vaccine manufacturing protocols to stimulate DC maturation (60). DCs are composed of subsets that differ in their phenotype, localization, and function. DLL4 + DC promote CD4^+^ T cell effector response. Blocking DLL4 causes a dramatic reduction of inflammatory T cell responses (60). Gentle et al. (59) demonstrated that DC stimulated concurrently with both Notch and TLR ligands have a distinct cytokine profile and are more pro-inflammatory compared with DCs stimulated with either ligand alone. This effect appears to be mediated by non-canonical Notch signaling (61, 62). Non-canonical Notch signaling regulates various pathways in cancer and immune cells (59). In DC, PI3kinase stimulated by membrane-bound Notch modulates the response to pro-inflammatory signals (59).

In summary, Notch signals play important roles in DC maturation and activity. Canonical and non-canonical Notch signaling are involved. In most cases, Notch activity seems to promote DC maturation and function, but pDC may be an exception. Strategies leading to Notch activation in DC may enhance the effectiveness of DC-based cancer immunotherapy strategies.

## Notch and TAMs

Macrophages are a multifunctional and heterogeneous cell population, which can originate from embryonic precursor cells within a tissue or derive from HSCs *via* the myelomonocytic lineage (63). They can function as phagocytes, APC, and modulators of innate and adaptive immune responses, tissue remodeling, and inflammation. Macrophages are phenotypically plastic, and at least in animal models two distinct polarization pathways have been identified: classic activation-M1 macrophages and alternative activation-M2 macrophages (64, 65). M1-macrophages are polarized and activated by interferon-γ and lipopolysaccharide. They are specialized in innate immune responses against intracellular pathogens. TLR receptors such as TLR4 in M1 macrophages trigger the activation of NF-κB, AP-1, and STAT1 and promote the release of pro-inflammatory cytokines such as IL1, TNFα, IL-12, IL-1, IL-6, IFNγ, and chemokines CCL2 and CXCL10 (66). M2 macrophages secrete anti-inflammatory cytokines such as IL-10 and TGF-β. These cells limit tissue damage caused by inflammation and promote tissue repair and remodeling. Their effects on the adaptive immune system are more complex, including activation and inhibition (67). Importantly, the M1 and M2 polarization states are not irreversible. They can be considered phenotypic manifestations of biological plasticity, and intermediate phenotypes are possible. Additional macrophage subpopulations are emerging (68) whose roles in cancer are still unclear.

Tumor-associated macrophages are important components of the tumor microenvironment (69). TAMs tend to acquire an M2-phenotype. Recent studies have shown that TAMs can originate either from resident tissue macrophages or from tumor-infiltrating monocytes (67). Studies in patient samples and animal models reveal that TAMs can promote tumor growth by modulating angiogenesis, remodeling the extracellular matrix, providing a niche for cancer stem cells, as well as directly enhancing invasion and metastasis (70–72). High numbers of TAMs are linked to poor prognosis in cancer and associated with increased angiogenesis, enhanced tumor cell invasion, and suppression of adaptive antitumor immunity (73, 74). In basal-like breast cancer, TAMs are associated with poor clinical outcomes (75).

Notch signals play important roles in the differentiation, polarization, and activation of macrophages. In general, Notch signaling mediators are upregulated in activated macrophages (76–80). Wang et al. reported that Notch signaling modulated the M1 or M2 polarization of macrophages in antitumor immune response (81). M2-like TAMs have decreased Notch activity. Activation of Notch signaling promoted an M1 phenotype, secretion of IL-12, and enhanced tumor immunity. These authors showed that canonical CSL/RBP-J-mediated Notch signaling modulates the M1 versus M2 polarization through SOCS3 (81). Xu et al. showed that Notch1 enhances the M1 polarization of inflammatory macrophages through canonical and mitochondrial signaling, whereby Notch1 NICD induces CSL-mediated expression of mitochondrial genes but also associates with mitochondria and modulates metabolic activity and mitochondrial genome expression (82).

An elegant study by the Reedijk group showed that Notch signaling in tumor cells regulates the expression of pro-inflammatory cytokines, IL1β and CCL2, and induced the recruitment of TAM (83). In addition, these authors found that Notch regulates TGFβ-mediated activation of tumor cells by TAMs, suggesting a paracrine loop between TAMs and cancer cells mediated by Notch signals. These authors found a strong association between Notch activation, IL1β and CCL2 production, macrophages infiltration in basal-like breast cancer (83). Zhang et al. analyzed patient samples of invasive micropapillary carcinoma of the breast and proposed that Jagged1-modulated TAM infiltration is associated with poor prognosis (84). Liu et al. found Jagged1 expression is associated with high stromal M2-like TAM and with reduced disease-free and overall survival in primary breast tumor tissues (85). Interestingly, they also found higher M2-like TAM infiltration in metastatic lesions than in primary tumor of patients with aromatase inhibitor resistant cancers. They concluded that Jagged1 promotes aromatase inhibitor resistance by inducing TAM differentiation in breast cancer patients (85). Tanase et al. proposed that TAM and Notch signaling cooperate in reprogramming the glioma stem cell niche, providing protection and support for glioma stem cells (86). Guo and Gonzalez-Perez described a novel crosstalk between Notch, IL-1, and leptin that induces angiogenesis in breast cancer (87). In their working model, leptin stimulates receptor and ligand expression in breast cancer cells. This phenomenon is dependent on IL-1 signaling. In turn, Notch contributes to the expression of VEGF/VEGFR2 and thus promotes angiogenesis. In this model, IL-1 produced by inflammatory cells such as TAM would enhance leptin-promoted Notch signaling. This crosstalk would be of particular importance in obesity-associated cancers, as leptin is increased in obese patients. Low-grade systemic chronic inflammation in obesity has been proposed to involve M1 macrophages (88). In this case, systemic production of pro-inflammatory cytokines such as IL-1 by M1 macrophages would promote tumor growth at least in part through Notch.

A recent study demonstrated that miR-148a-3p acts downstream of Notch to promote the differentiation of monocytes into macrophages (89). Following Notch activation, miR-148a-3p promoted M1 but inhibited M2 polarization of macrophages. In a transgenic mouse model, conditional overexpression of NICD had no effect of TAM differentiation, but abrogated TAM functions (90). The same study identified miR-125a as a novel downstream mediator of Notch signaling. A miR-125a mimetic increased the phagocytic activity of macrophages and suppressed tumor growth by remodeling tumor microenvironment (90).

In conclusion, Notch signaling participates in the polarization of macrophages and modulates their activity. Furthermore, cytokines produced by macrophages stimulate Notch in cancer cells, and paracrine loops between macrophages and cancer cells can promote tumor survival.

## Concluding Remarks

After decades of preclinical studies with only anecdotal clinical successes, cancer immunotherapy has entered a new phase. Immune checkpoint blockade therapy is one of the most radical innovations in clinical oncology in recent years (91). The FDA approval of CAR-T cell therapy in 2017 was another momentous development (92). However, despite the power of these approaches, there remain plenty of challenges to their clinical application on a large scale. For instance, cancers with low mutational burden are less likely to respond to immunotherapy, perhaps due to their limited antigen repertoire (93). The identification of patients and tumors most likely to respond to immunotherapy through precision medicine approaches is one of the most promising strategies to enhance the impact of cancer immunotherapy. In 2017, in a landmark development, the U.S. FDA granted accelerated approval of an anti-PD-1 antibody to treat patients whose cancers show microsatellite instability or somatic defects in DNA mismatch repair. This was the first FDA approval of an anti-neoplastic agent based not on anatomical cancer location or tumor type but on genomic biomarkers.

**Figure 2:**
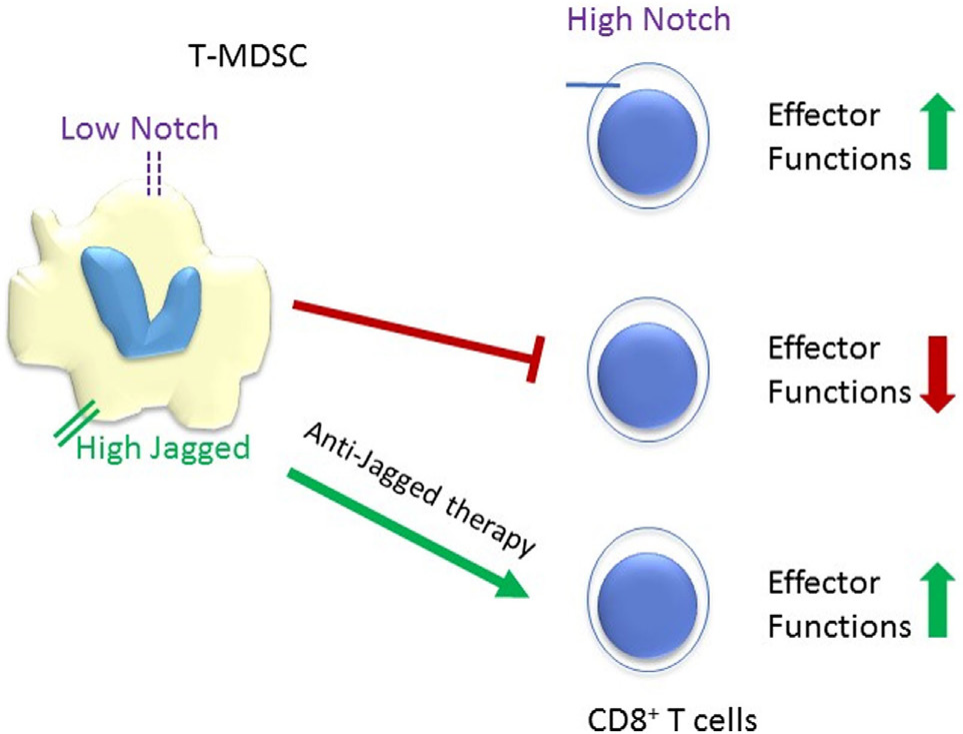
Schematic representation of the reciprocal responses of tumor-infiltrating MDSC (T-MDSC) and CD8^+^ T cells to Notch signaling. High Notch signaling promotes CD8^+^ T cells effector functions, while low Notch signaling spurs T-MDSC. Tumor microenvironments upregulate the expression of Notch ligand Jagged on T-MDSC and anti-Jagged therapy overcome tumor-induced T cells tolerance (36). It is unclear whether Jagged expressed in myeloid-derived suppressor cell (MDSC) competes with DLL ligands for Notch1 and Notch2 in CD8 T-cells, or potentially with TCR-induced ligand-independent activation. However, blockade of Jagged1 and 2 in MDSC restores CD8 effector functions.

Immune suppression by TME myeloid cells is one of the main challenges to the large scale application of cancer immunotherapy. The intricate crosstalk between systemic inflammation, myeloid cells in tumor microenvironment, the cancer cell themselves, and multiple lymphocyte subpopulations modulates tumor immunity. Notch signaling plays multiple roles in this crosstalk (Figure 2), and potential therapeutic applications of Notch modulation in immunotherapy have shown significant promise. Among these, the inhibition of MDSC functions by Jagged antibodies and the enhancement of CD8 resistance to MDSC by CD8 T cell-selective Notch activation appear particularly attractive. Another attractive target is DLL4. Tumor-infiltrating myeloid cells activate Dll4/Notch/TGF-β signaling to drive malignant progression (94). A human DLL4 monoclonal antibody by Oncomed Pharmaceuticals is presently in a phase Ib clinical trial in combination with anti PD-1. Combination cancer immunotherapy, particularly targeting the interaction between myeloid cells and T cells in the tumor microenvironment, is a potentially attractive strategy for Notch-targeted drugs and biologics.

## Author Contributions

FH, SM, and DU wrote different sections of this manuscript. PR, BO, TG, and LMM provided critical input. LM reviewed and edited the draft, and wrote the final version of the manuscript.

## Conflict of Interest Statement

The authors declare that the research was conducted in the absence of any commercial or financial relationships that could be construed as a potential conflict of interest.
